# Genome-wide identification and expression analysis of *TPP* gene family under salt stress in peanut (*Arachis hypogaea* L.)

**DOI:** 10.1371/journal.pone.0305730

**Published:** 2024-07-18

**Authors:** Yanfeng Zhang, Minxuan Cao, Qiuzhi Li, Fagang Yu

**Affiliations:** 1 College of Agriculture, Nanjing Agricultural University, Nanjing, Jiangsu, China; 2 College of Agriculture & Biotechnology, Zhejiang University, Hangzhou, Zhejiang, China; 3 Liaocheng Academy of Agricultural Sciences, Liaocheng, Shandong, China; Agri Biotech Foundation and Retired Professor, University of Hyderabad, INDIA

## Abstract

Trehalose-6-phosphate phosphatase (TPP), a key enzyme for trehalose biosynthesis in plants, plays a pivotal role in the growth and development of higher plants, as well as their adaptations to various abiotic stresses. Employing bioinformatics techniques, 45 *TPP* genes distributed across 17 chromosomes were identified with conserved Trehalose-PPase domains in the peanut genome, aiming to screen those involved in salt tolerance. Collinearity analysis showed that 22 *TPP* genes from peanut formed homologous gene pairs with 9 *TPP* genes from *Arabidopsis* and 31 *TPP* genes from soybean, respectively. Analysis of cis-acting elements in the promoters revealed the presence of multiple hormone- and abiotic stress-responsive elements in the promoter regions of *AhTPP*s. Expression pattern analysis showed that members of the *TPP* gene family in peanut responded significantly to various abiotic stresses, including low temperature, drought, and nitrogen deficiency, and exhibited certain tissue specificity. Salt stress significantly upregulated *AhTPP*s, with a higher number of responsive genes observed at the seedling stage compared to the podding stage. The intuitive physiological effect was reflected in the significantly higher accumulation of trehalose content in the leaves of plants under salt stress compared to the control. These findings indicate that the *TPP* gene family plays a crucial role in peanut’s response to abiotic stresses, laying the foundation for further functional studies and utilization of these genes.

## Introduction

Peanut (*Arachis hypogaea* L.) is a global oilseed and economic crop. Not only does it serve as a crucial source of high-quality vegetable oils and proteins, it also serves as a primary raw material for the production of high-quality food products. The peanut industry holds significant development potential [1, 2]. Salt stress is a critical factor that restricts plant growth and development, and soil salinization represents a serious environmental challenge for global irrigated agriculture. It is also the main driver of land degradation, leading to soil compaction, fertility loss, and exacerbated soil erosion. The reasons for soil salinization include improper irrigation, vegetation damage, and seawater intrusion. Currently, the global saline-alkali land area measures approximately 954 million hectares, and spans various continents and subcontinent regions [3]. The total area of saline-alkali land in China also stands at 99.13 million hectares, accounting for about 10% of the country’s landmass [3, 4]. Almost all peanut species belong to glycophytes or non-halophytes and display sensitivity to Na^+^. When the soil salinity exceeds 0.3%, it will affect the growth and development of peanuts, cause growth obstacles, and seriously reduce peanut yield [5]. In production, the threshold for soil salt content to ensure peanut emergence is about 0.45%, and exceeding this concentration may result in the inability to emerge [6].

Trehalose is a non-reducing disaccharide widely present in plants, bacteria, and invertebrates. It not only serves as a carbon storage and transportation mechanism within organisms but also acts as a protective agent that enhances resistance to adverse environments [7, 8]. In plants, trehalose synthesis occurs through a two-step enzymatic process. Initially, the enzyme trehalose-6-phosphate synthase (TPS) catalyzes the conversion of uridine diphosphate glucose and glucose-6-phosphate into trehalose-6-phosphate (T6P). Subsequently, trehalose-6-phosphate phosphatase (TPP) catalyzes the dephosphorylation of T6P, ultimately producing trehalose. It has been established through research that the accumulation of trehalose in organisms is closely related to stress. Under heat shock conditions, the trehalose content in *Saccharomyces cerevisiae* reached 30% of cell dry weight. Under high temperatures, yeast strains that knocked out the *TPS1* gene were difficult to grow, while strains that overexpressed *TPS1* grew well [9, 10]. The increase in trehalose content of *Arabidopsis* enhances its tolerance to salt stress [11]. Furthermore, trehalose-6-phosphate synthase plays a crucial role in regulating carbohydrate metabolism, growth, development, and stress response. Compared with non-transgenic rice, transgenic rice overexpressing the *TPS* gene maintained better growth under high salt, drought, and low-temperature conditions [12]. The aforementioned research indicates that trehalose and its metabolite T6P are involved in biological signal regulation, and they may serve as important metabolic regulators, coordinating multiple metabolic pathways in plants.

In higher plants, *TPP* genes are distributed on different chromosomes in the form of a gene family, mainly characterized by the presence of a conserved Trehalose-PPase domain [13–15]. With the rapid development of whole genome sequencing technology in plants, the *TPP* gene family has been identified in numerous species and has been confirmed to be involved in the abiotic stress response of crops such as wheat, rice, and maize [16–18]. As an important economic crop, research on the *TPP* gene family based on the genome level remains limited in peanut. Genus Arachis contains 81 species, most of which are diploid (2n = 2x = 20), while the cultivated peanut (*Arachis hypogaea* L.) is allotetraploid (AABB, 2n = 4x = 40). Cytogenetic, phylogeographic and molecular-biological evidence suggested that allotetraploid *A*. *hypogaea* might be formed by the hybridization of diploid *A*. *duranensis* (AA) and *A*. *ipaensis* (BB), whose genome size is twice that of wild diploid [19–21]. The relatively large genome increases the genetic complexity of peanut. Here, we utilized bioinformatics techniques to identify the members of *TPP* gene family of peanut, and analyzed their gene structure, chromosome localization, cis-acting elements, and expression patterns at the genomic level, to provide a theoretical basis for further exploring the specific biological functions of this gene family and the molecular mechanism of abiotic stress-induced trehalose accumulation in peanut.

## Materials and methods

### Plant materials and treatment

The study focused on two peanut varieties, Huayu963 (salt-resistant) and Weihua22 (salt-sensitive), with a high oleic acid content and a large cultivation area in China. The peanut seeds were immersed in distilled water for 4 hours, transferred to culture dishes containing moist filter paper, and subsequently germinated in the dark at 28°C for 2 days. The germinated peanut seeds were planted in polyethylene plastic pots with a closed bottom. The plastic pot had a diameter of 24 cm, a height of 25 cm, and was filled with seven kilograms of soil per pot. Three seedlings were left in each pot. The soil used in the experiment was yellow-brown soil. All treatments adopted standard and consistent water & fertilizer management. The potted experiment was conducted in a greenhouse at Baima Experimental Base of Nanjing Agricultural University in Lishui District, Nanjing City, Jiangsu Province, China (119°09’E, 31°35’N). The tested peanut varieties were provided by Liaocheng Academy of Agricultural Sciences.

The treatments of this experiment were divided into control (C) and salt stress (S) groups. The control treatment did not receive any sodium chloride. The salt stress treatment involved applying 2 L of 0.24 mol/L sodium chloride solution per pot before sowing, simulating a soil salt content of 4‰, which is considered stressful for production. The experiment utilized a randomized block design, with three replicates for each treatment. During the seedling and podding stages, six leaves of similar size were collected from different plants in each replicate, quick-frozen in liquid nitrogen, and stored at -80°C for subsequent experimental analysis.

### Phenotypic observation and physiological index measurements

Chlorophyll Fluorescence Imager (CF Imager, Ecotek, Beijing, China) was used to assess various parameters of peanut leaves from different treatments during the podding stage, including the PSII excitation energy capture efficiency (XE, expressed as Fv’/Fm’), the electron transport rate (EF, expressed as Fq’/Fv’), the PSII actual photochemical quantum efficiency (OE, expressed as Fq’/Fm’), and the non-photochemical quenching coefficient (NPQ). FluorCam (Photon Systems Instruments, Czech) was used to observe chlorophyll fluorescence parameters of whole peanut plants from different treatments during the podding stage.

The determination of trehalose content was carried out using the anthrone-sulfuric acid method [22]. 0.5 grams of peanut leaf dry sample powder was accurately weighed, mixed with 5 mL of 0.5 mol/L trichloroacetic acid solution, and ground in an ice water bath. After being shaken at 0°C for 12 h, the mixture was centrifuged at 6000 r/min for 10 min. We took 2 mL of the supernatant, added 4 mL of 0.2% anthrone-sulfuric acid mixed reagent, heated it in boiling water for 5 minutes, cooled it down, and measured its absorbance at a wavelength of 590 nm.

### Identification and phylogenetic analysis of *TPP* gene family in peanut

The genomic data information for peanut (Ahypogaea_530_v1.0.fa, Ahypogaea_530_v1.0.gene.gff3, Ahypogaea_530_v1.0.cds.fa, Ahypogaea_530_v1.0.protein.fa), *Arabidopsis* (Arabidopsis_thaliana.TAIR10.41.gff3, Arabidopsis_thaliana.TAIR10.cds.all.fa), soybean (Glycine_max.Glycine_max_v2.1.57.chr.gff3, Glycine_max.Glycine_max_v2.1.cds.all.fa), and rice (Oryza_sativa.IRGSP_1.0.cds.all.fa, Oryza_sativa.IRGSP_1.0.51.gff3) was downloaded from the Ensemble database (http://plants.ensembl.org/index.html). The reference genome of cultivated peanut (Arachis hypogaea L.) used in this study was released by the team of Dr. David J. Bertioli. Taking A. hypogaea cv. Tifrunner as the material, using a whole-genome shotgun sequencing strategy, they completed the precise assembly of 20 chromosomes in the A and B subgenomes of allotetraploid peanut cultivars, so as to obtain the high-quality genome sequence of cultivated peanut [23]. The HMM profile for the trehalose-PPase domain (PF02358) was downloaded from the Pfam database (https://www.ebi.ac.uk/interpro/entry/pfam). Perl script was used to preliminarily screen protein sequences containing the trehalose-PPase domain in the peanut genome. Subsequently, HMMER 3.0 software was employed to construct a hidden Markov model specific to the peanut’s *TPP* gene family. This newly constructed model was then used for secondary retrieval in the peanut genome, with validation provided by the Pfam database and CDD tools (http://www.ncbi.nlm.gov/cdd).

Using MEGA 11 software, we conducted a comparative analysis of the *TPP* protein sequences identified in peanut with those previously characterized in *Arabidopsis*, rice, and soybean [24–26], and the result was visualized through a phylogenetic tree based on neighbor-joining method. The phylogenetic tree was refined using the evolview website (https://evolgenius.info//evolview-v2/#login). TBtools software was utilized to efficiently calculate the physicochemical properties of proteins in batches. WoLF PSORT website (https://www.genscript.com/wolf-psort.html) was used to predict subcellular localization information of proteins.

### Physical localization of *TPP* gene family on chromosomes in peanut

To determine the positions of *TPP* gene family members on the peanut chromosome, we retrieved the gene and chromosome information from the gff file of the peanut genome. Mapchart 2.32 was used to draw a distribution map of genes on chromosomes according to the length ratios of relevant genes and chromosomes.

### Prediction of conserved motifs and gene structural analysis of *AhTPP*s

Motifs in protein sequences of *AhTPP*s were predicted using MEME 5.5.3 online program (http://meme-suite.org). After obtaining the information on *AhTPP*s from the gff file, we input the relevant information into TBtools software, and draw a combined diagram of gene structure and conserved motifs.

### Protein multiple sequence alignment and analysis of the cis-acting elements of the *TPP* gene family members in peanut

We used MEGA 11 for protein multi-sequence alignment, and imported the aligned fasta file into GeneDoc software to analyze conserved protein domains.

Based on the sequences of identified target genes, we extracted 1500 bp of upstream genome sequences from *AhTPP*s and used the PlantCARE database (http://bioinformatics.psb.ugent.be/webtools/plantcare/html/) to predict and analyze the cis-acting elements within promoter regions.

### Collinearity analysis of *TPP* gene family members within the peanut genome and comparative genomic analysis of homologous relationships among different species

We used MCScanX on the biolinux system to conduct a collinearity analysis of the peanut genome and employed the Circos tool for enhanced visualization.

We used the Python version of MCscan to perform collinearity analysis between the genomes of peanut, *Arabidopsis*, rice, and soybean. The image beautification method was predicated on the codes provided on the official website (https://github.com/tanghaibao/jcvi/wiki/MCscan-(Python-version)).

### Expression pattern analysis of *TPP* gene family in peanut under nitrogen deficiency, drought, and cold stress

We downloaded four sets of peanut’s transcriptome data under nitrogen deficiency, drought, and cold stress from supplementary datasets of four publicly published SCI paper to analyze the expression patterns of *AhTPP*s under different abiotic stresses [27–30]. R program was used to draw expression heatmaps. The FPKM (Fragments Per Kilobase of exon model per Million mapped fragments) matrixes of differential genes were extracted, and heatmaps were created through the Pheatmap package.

### Analysis of gene expression by real-time quantitative RT-PCR

Total RNA was extracted from the peanut leaves using Trizol Reagent (Invitrogen, San Diego, CA, USA) according to the manufacturer’s instructions. The first strand of cDNAs was synthesized from 1 μg of DNaseI-treated total RNA using HiScript III RT SuperMix reverse transcriptase kit (Vazyme Biotech Co., Ltd, China). Quantitative RT-PCR (qRT-PCR) assays were performed on the Bio-Rad CFX96 RT-PCR Detection system (Bio-Rad, Hercules, CA, USA) using ChamQ SYBR qPCR Master Mix (Vazyme Biotech Co., Ltd, China). The relative transcript levels among different samples were quantified by the 2^-ΔΔCt^ method [31], using *β-Actin* as a reference gene for normalization [32]. The expression level of treatment C was used as a control and the relative expression level of treatment S was determined relative to the control.

## Results and analysis

### The effect of salt stress on the peanut growth and trehalose content in leaves

Salt stress had a significant impact on the growth status of two peanut varieties during the seedling stage, mainly manifested as delayed nutritional growth, stunted and weak plants, and reduced survival rate (S1 Fig). The salt-sensitive variety (Weihua22) displayed a distinct stress phenotype, with seedlings gradually succumbing to death after germination. Compared to the 89% survival rate of the salt-resistant variety (Huayu963), the survival rate of the salt-sensitive variety under salt stress was only 56%.

**Fig 1:**
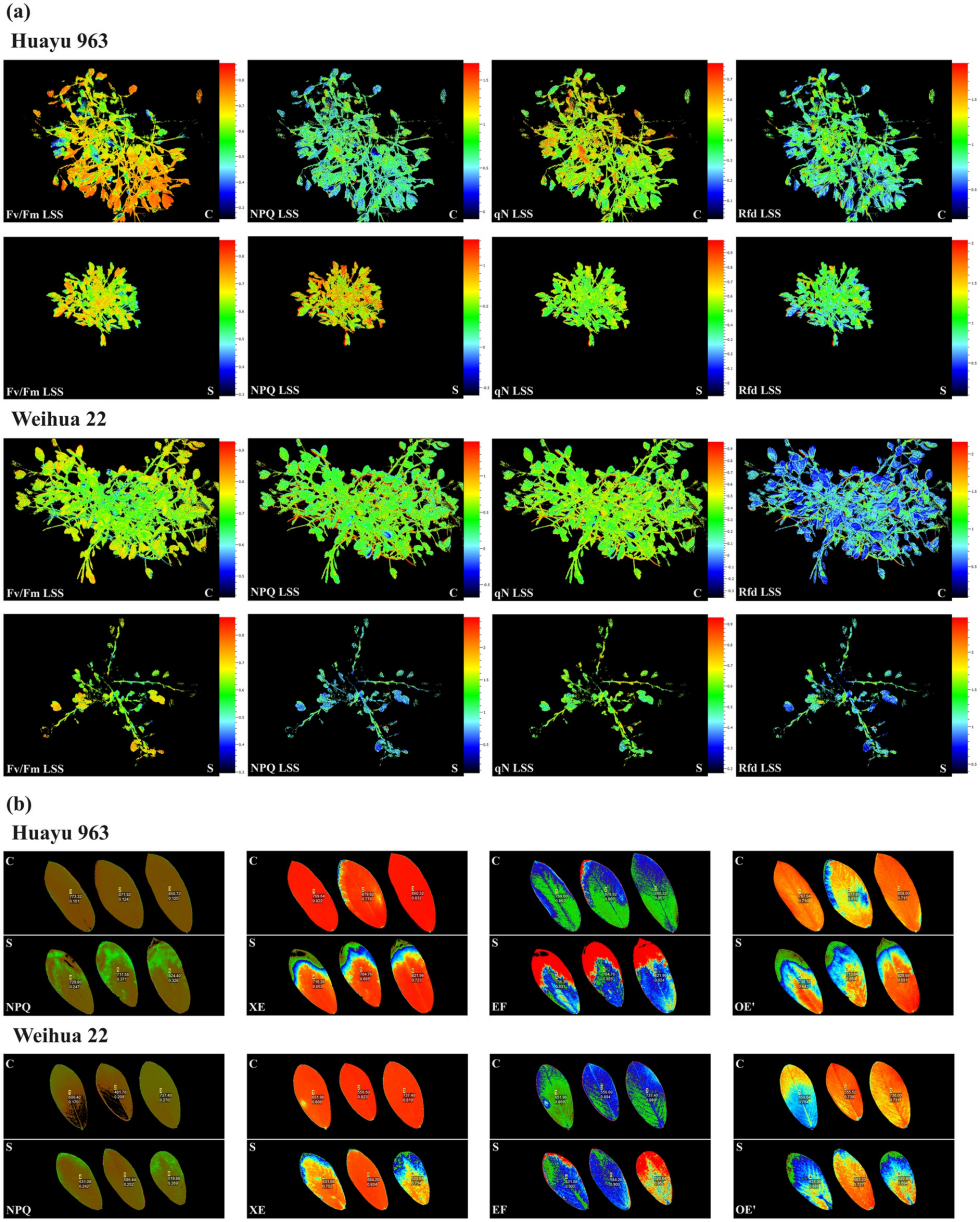
Fluorescence parameters of the whole plant (a) and single leaf (b) of two peanut varieties during podding stage under salt treatment (S) and normal cultivation treatment (C) in the chlorophyll fluorescence imaging system. Fv/Fm Lss means PSII quantum yield of light adapted sample at steady-state. NPQ_Lss means steady-state non-photochemical quenching in light. qN_Lss means non-photochemical quenching at steady-state. Rfd_Lss means fluorescence decline ratio in steady-state. XE means the efficiency of excitation energy capture by open PSII reaction centers. EF means the coefficient of photochemical quenching. OE’ means the actual photochemical efficiency of PSII in light.

During the podding stage, we observed photosynthetic fluorescence parameters of the whole plants of two peanut varieties and found that salt stress significantly reduced parameters such as Fv/Fm and qN (Fig 1a). In addition, we also conducted an observation experiment on the leaves and found that under salt stress, parameters such as XE and OE’ of leaves significantly decreased (Fig 1b). It is worth noting that an increase in NPQ was observed in whole plants or individual leaves under salt stress. The above results indicated that salt stress caused damage to the PSII reaction center in peanut leaves, inhibited PSII photochemical activity, reduced PSII primary light energy conversion efficiency, suppressed PSII potential activity, and disrupted the primary reaction process of peanut photosynthesis. Nevertheless, it also increased heat dissipation and provided light protection for PSII. In summary, high soil salinity has a long-term and persistent adverse impact on peanut production, affecting both nutritional and reproductive growth stages.

The measurement results of trehalose content in the leaves of two peanut varieties during the seedling and podding stages showed that, except for Weihua22 during the podding stage which did not show significant differences between treatments, the trehalose content in leaves of other stages under salt stress was significantly higher than that of the control treatment, and the interaction effect between the variety and the growth stage was significant (Table 1). The correlation analysis between trehalose content and photosynthesis indices under control and salt stress at the seedling stage of peanut showed that the trehalose content in leaves was relatively low and had no significant correlation with photosynthetic indices under normal cultivation conditions, while it was significantly increased and had a significant positive correlation with photosynthetic indices under salt stress (S2 Fig). These findings suggest that the accumulation of trehalose has a positive effect on peanut adaptation to salt environments.

**Table 1:** The effect of salt stress on trehalose content in leaves of two peanut varieties during the seedling and podding stages based on ANOVA.

Variety	Growth stage	Treatment	Trehalose content (μg/g)
Huayu963	Seedling stage	C	1.846±0.006 ^d^
		S	1.872±0.018 ^c^
	Podding stage	C	1.895±0.001 ^b^
		S	1.916±0.007 ^a^
Weihua22	Seedling stage	C	1.806±0.004 ^c^
		S	1.829±0.009 ^b^
	Podding stage	C	1.889±0.001 ^a^
		S	1.899±0.003 ^a^
F-value		V	71.85 **
		G	340.13 **
		T	29.06 **
		V×G	15.78 **
		V×T	0.99
		G×T	4.09
		V×G×T	0.45

Different letters represent significant differences. The superscript of the largest value among treatments is marked as “a”, the superscript of the treatment with statistically significant reduction compared with treatment “a” is marked as “b”, the superscript of the treatment with statistically significant reduction compared with treatment “b” is marked as “c”, and the superscript of the treatment with statistically significant reduction compared with treatment “c” is marked as “d”. If the differences between treatments are not statistically significant, the superscripts remain the same letter.

** indicates significance at 0.01 level.

### Identification and phylogenetic analysis of TPP gene family members in peanut

**Fig 2:**
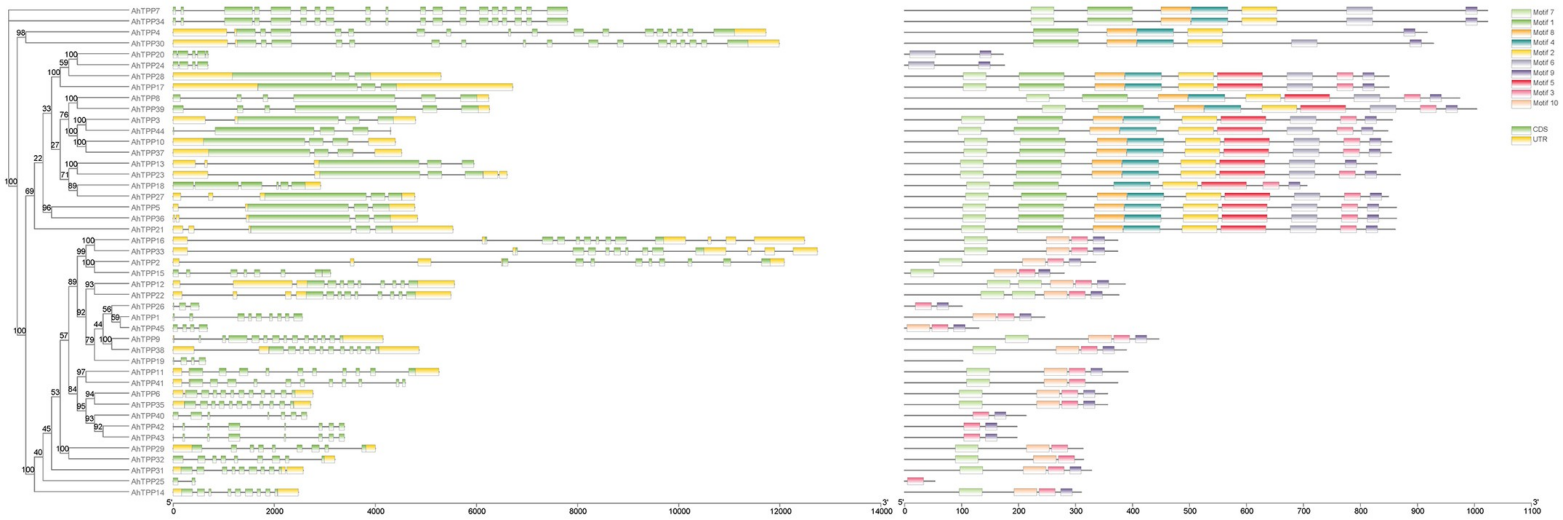
Analysis of gene structures and conserved motifs in the phylogenetic tree of 45 *AhTPP*s. The phylogenetic tree was constructed using the NJ method. Exons and introns are shown as rectangles and lines, respectively. Ten conserved motifs predicted in proteins coded by *AhTPP*s are shown as differently colored boxes.

45 *AhTPP*s were identified from the peanut genome through comprehensive screening and were assigned the names from *AhTPP1* to *AhTPP45* (Table 2). Analysis revealed a notable variation in the total length of *TPP* gene family members of peanut, with *AhTPP33* having the longest total length and *AhTPP25* having the shortest total length. For the CDS sequence, *AhTPP7* and *AhTPP34* have the longest length, both reaching 3069 bp, while *AhTPP25* has the shortest length, only 159 bp. Gene structure analysis shows that all family members contain exons and introns, but there is a significant difference in the number of exons, ranging from a maximum of 19 to a minimum of 2 (Fig 2). The number of amino acids ranges from 52 to 1022. The isoelectric point range is from 4.24 to 9.48. The relative molecular weight range is from 6.11 to 116.17 KD. Except for the protein encoded by *AhTPP45*, which was predicted to be hydrophobic, all other proteins are hydrophilic. Subcellular localization prediction indicated that 14 members were located in the cycloplast, 2 members in the cytoskeleton, 13 members in the nucleus, 14 members in the chloroplast, and 2 members in the endoplasmic reticulum. It can be inferred that proteins encoded by the 45 *AhTPP*s may perform diverse biological functions in different organelles of peanut.

**Table 2:** Physicochemical properties of *TPP* gene family proteins in *Arachis hypogaea* L.

Gene name	Gene ID	CDS (bp)	Number of amino acids	Molecular weight	PI	GRAVY	Subcellular localization
AhTPP1	arahy.Tifrunner.gnm1.ann1.8AAC5A	738	245	27864.94	5.44	-0.203	Cytoplasm
AhTPP2	arahy.Tifrunner.gnm1.ann1.U769C2	1005	334	38130.21	5.49	-0.375	Cytoskeleton
AhTPP3	arahy.Tifrunner.gnm1.ann1.9VZ5EJ	2568	855	96691.62	5.69	-0.196	Cytoplasm
AhTPP4	arahy.Tifrunner.gnm1.ann1.YY1VWG	2751	916	103010.51	7.36	-0.349	Nucleus
AhTPP5	arahy.Tifrunner.gnm1.ann1.IE8W25	2589	862	97108.04	5.88	-0.189	Chloroplast
AhTPP6	arahy.Tifrunner.gnm1.ann1.FJ2B1F	1068	355	39946.89	9.22	-0.393	Cytoplasm
AhTPP7	arahy.Tifrunner.gnm1.ann1.PK6QLT	3069	1022	116170.19	8.31	-0.296	Cytoplasm
AhTPP8	arahy.Tifrunner.gnm1.ann1.FTDL1P	2922	973	109322.73	5.81	-0.26	Nucleus
AhTPP9	arahy.Tifrunner.gnm1.ann1.CK7SVG	1338	445	49874.19	7.59	-0.256	Cytoplasm
AhTPP10	arahy.Tifrunner.gnm1.ann1.Q3GMD8	2565	854	96681.96	5.77	-0.188	Nucleus
AhTPP11	arahy.Tifrunner.gnm1.ann1.SS1VDK	1176	391	43915.47	9.04	-0.353	Chloroplast
AhTPP12	arahy.Tifrunner.gnm1.ann1.51Y4LP	1161	386	43296.31	6.22	-0.381	Nucleus
AhTPP13	arahy.Tifrunner.gnm1.ann1.PWAA2D	2487	829	94050.06	5.82	-0.236	Chloroplast
AhTPP14	arahy.Tifrunner.gnm1.ann1.B1753N	930	309	34895.17	9.07	-0.348	Endoplasmic reticulum
AhTPP15	arahy.Tifrunner.gnm1.ann1.B2J19D	840	279	31689.36	7.79	-0.295	Cytoskeleton
AhTPP16	arahy.Tifrunner.gnm1.ann1.0FY2NM	1122	373	42405.02	9.22	-0.332	Chloroplast
AhTPP17	arahy.Tifrunner.gnm1.ann1.G88L7W	2550	849	96026.65	5.81	-0.226	Chloroplast
AhTPP18	arahy.Tifrunner.gnm1.ann1.93VR75	2118	705	80392	6.89	-0.283	Chloroplast
AhTPP19	arahy.Tifrunner.gnm1.ann1.E7HJTX	306	101	11442.06	6.27	-0.4	Cytoplasm
AhTPP20	arahy.Tifrunner.gnm1.ann1.G4A8LI	519	172	19717.64	5.81	-0.32	Cytoplasm
AhTPP21	arahy.Tifrunner.gnm1.ann1.AKPI0I	2583	860	96986.1	6.11	-0.175	Cytoplasm
AhTPP22	arahy.Tifrunner.gnm1.ann1.5Q8BGE	1128	375	42051.76	6.03	-0.41	Nucleus
AhTPP23	arahy.Tifrunner.gnm1.ann1.FZMU71	2610	869	98141.75	5.73	-0.225	Chloroplast
AhTPP24	arahy.Tifrunner.gnm1.ann1.4K8ZJG	525	174	19474.37	5.36	-0.11	Cytoplasm
AhTPP25	arahy.Tifrunner.gnm1.ann1.GS1Q1E	159	52	6112.71	4.24	-0.835	Chloroplast
AhTPP26	arahy.Tifrunner.gnm1.ann1.83R1N2	303	100	11240.15	4.54	0.613	Chloroplast
AhTPP27	arahy.Tifrunner.gnm1.ann1.A0DAR9	2547	848	96610.43	5.91	-0.221	Chloroplast
AhTPP28	arahy.Tifrunner.gnm1.ann1.1XW75L	2550	849	95996.62	5.81	-0.225	Chloroplast
AhTPP29	arahy.Tifrunner.gnm1.ann1.J3QXZL	939	312	35412.85	9.31	-0.366	Cytoplasm
AhTPP30	arahy.Tifrunner.gnm1.ann1.E48PAY	2784	927	104664.21	7.01	-0.383	Nucleus
AhTPP31	arahy.Tifrunner.gnm1.ann1.KW1U5A	984	327	36737.21	9.18	-0.378	Endoplasmic reticulum
AhTPP32	arahy.Tifrunner.gnm1.ann1.DP0G5T	942	313	35206.75	9.48	-0.296	Chloroplast
AhTPP33	arahy.Tifrunner.gnm1.ann1.G2WE73	1122	373	42431.04	9.33	-0.343	Chloroplast
AhTPP34	arahy.Tifrunner.gnm1.ann1.S5S9QI	3069	1022	116170.19	8.31	-0.296	Cytoplasm
AhTPP35	arahy.Tifrunner.gnm1.ann1.P5P8C7	1068	355	39933.8	9.08	-0.392	Cytoplasm
AhTPP36	arahy.Tifrunner.gnm1.ann1.CM68RF	2589	862	97165.09	5.79	-0.184	Nucleus
AhTPP37	arahy.Tifrunner.gnm1.ann1.FP7P7G	2562	853	96618.88	5.77	-0.195	Nucleus
AhTPP38	arahy.Tifrunner.gnm1.ann1.FIK9JS	1167	388	43213.37	6.57	-0.275	Cytoplasm
AhTPP39	arahy.Tifrunner.gnm1.ann1.RIH8EG	3012	1003	113229.45	6.22	-0.275	Nucleus
AhTPP40	arahy.Tifrunner.gnm1.ann1.EKX8HF	639	212	23489.3	5.09	-0.433	Nucleus
AhTPP41	arahy.Tifrunner.gnm1.ann1.S15BQI	1122	374	41950.96	9.12	-0.472	Chloroplast
AhTPP42	arahy.Tifrunner.gnm1.ann1.C51V2Y	591	196	22127.73	5.72	-0.706	Nucleus
AhTPP43	arahy.Tifrunner.gnm1.ann1.0S0AWB	591	196	22127.73	5.72	-0.706	Nucleus
AhTPP44	arahy.Tifrunner.gnm1.ann1.BL0RCS	2544	847	95865.65	5.6	-0.216	Nucleus
AhTPP45	arahy.Tifrunner.gnm1.ann1.7Q19EE	390	129	14541.95	5.86	0.117	Cytoplasm

**Fig 3:**
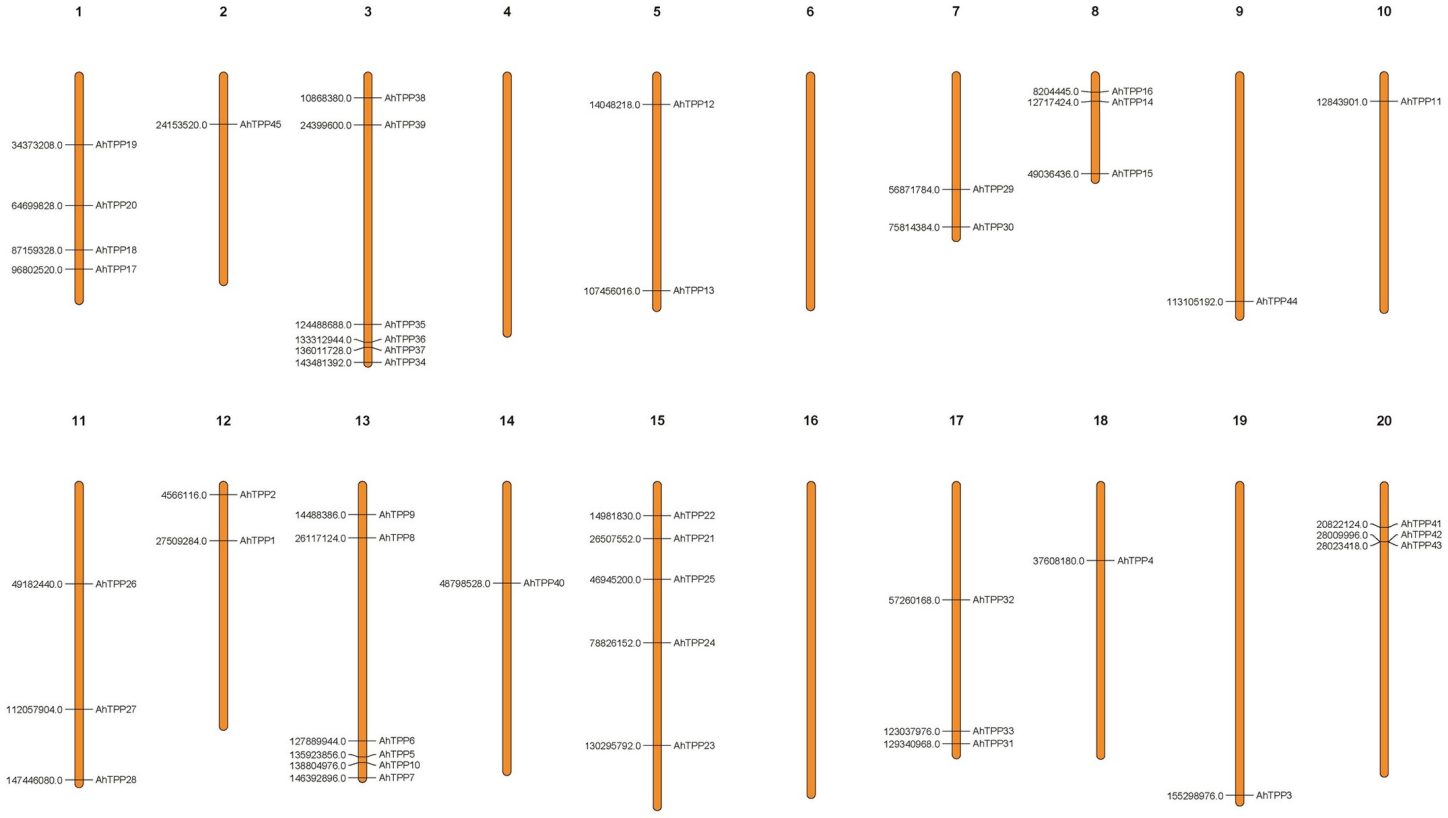
Chromosomal distribution of *TPP* genes in the peanut genome. The chromosome number and the specific location of genes on chromosomes are displayed in the figure.

45 *AhTPP*s are relatively evenly distributed on 17 chromosomes of peanut. Among them, chromosomes 4, 6, and 16 do not contain *TPP* genes. There are a large number of *TPP* genes on chromosomes 3 and 13, each with 6. The number of *TPP* genes on chromosomes 2, 9, 10, 14, 18, and 19 is relatively small, all with only one gene (Fig 3).

**Fig 4:**
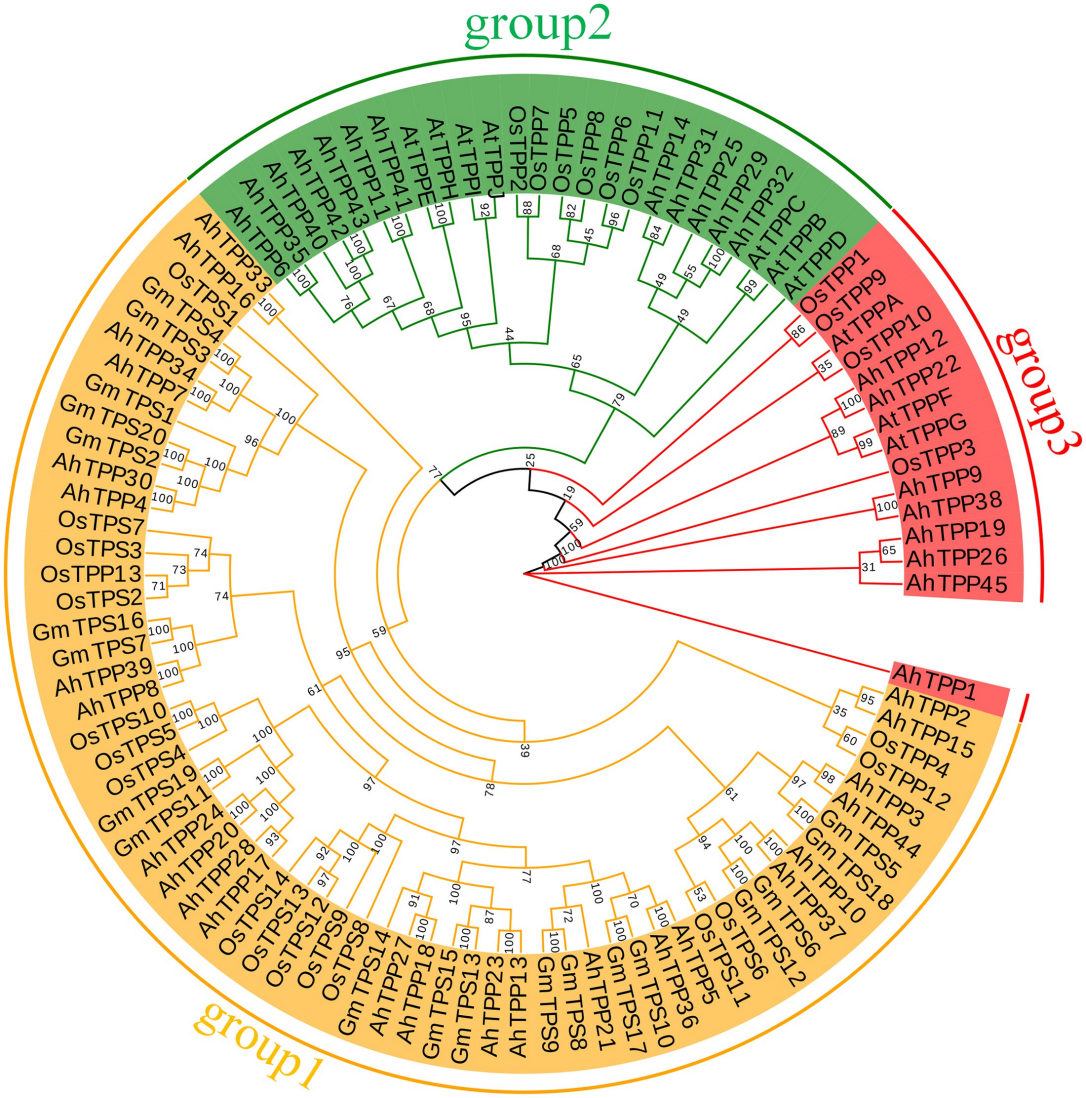
Systematic evolution of *TPP* proteins in *Arachis hypogaea*, *Glycine max*, *Oryza sativa*, and *Arabidopsis thaliana* by the neighbor-joining method.

To further understand the phylogenetic relationship of *TPP* genes, 102 protein sequences including monocotyledon (rice) and dicotyledon (*Arabidopsis*, soybean, peanut) were compared, and a phylogenetic tree was constructed (Fig 4). Phylogenetic analysis shows that *TPP* gene family members of peanut can be divided into three subgroups. The first subgroup has 25 members from peanut, and all genes from soybean are clustered in this group, indicating that the *AhTPP*s of this subgroup have high homology with soybeans. The second and third subgroups have 12 and 8 members from peanut, respectively. The genes from *Arabidopsis* are concentrated in the second and third subgroups, while rice genes are distributed in all subgroups.

### Motif analysis and multiple sequence alignment of proteins encoded by *AhTPP*s

**Fig 5:**
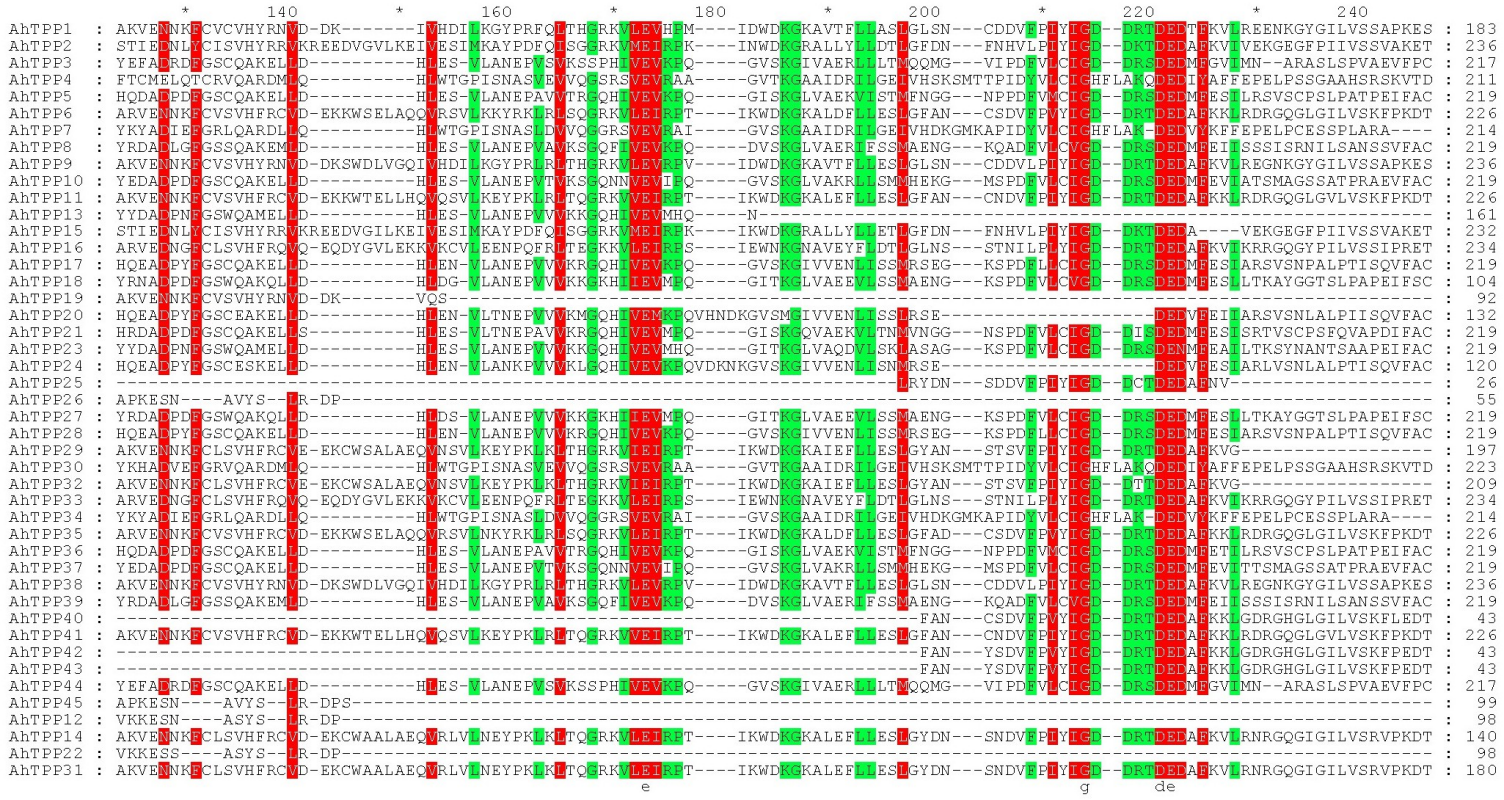
Multiple alignment of amino acid sequences of *AhTPP*s.

Through Pfam database and CDD tools validation, it was found that all 45 *AhTPP*s contain Trehalose-PPase (TPP, PF02358) domain, some members contain Glyco_transf_20 (TPS, PF00982) and Trehalose-PPase double domains. Using the MEME program for motif analysis of *AhTPP*s, it was found that *AhTPP*s have a total of 10 conserved motifs. The coverage rates of motif 3, motif 7, and motif 9 in the gene family are as high as 82%, 73%, and 89%, respectively, possessing high conservatism (Fig 2). The protein multi-sequence alignment of *AhTPP*s further supports the observation of high amino acid sequence similarity within these three motif regions (Fig 5).

### Analysis of cis-acting elements of *AhTPP*s

**Fig 6:**
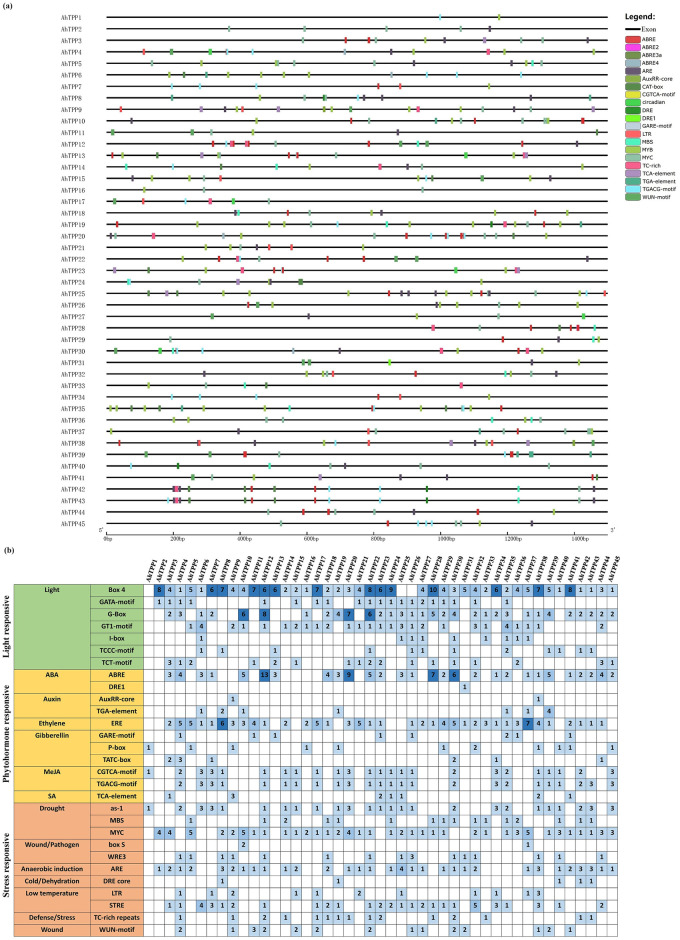
The distribution of cis-acting elements in promoters of the *TPP* gene family members in peanut (a) and statistics on the numbers of cis-acting elements related to major physiological processes (b).

The analysis of the cis-acting elements located within the 1500 bp regions of the upstream transcription start site of *AhTPP*s revealed that the majority of members possess more than ten cis-acting elements, with many of the elements being duplicated (Fig 6a). The cis-acting elements mainly include light responsive elements (Box 4, G-Box, GT1-motif, etc.), plant hormone responsive elements (ABRE, ERE, CGTCA-motif, etc.), and stress responsive elements (MYC, ARE, STRE, etc.) (Fig 6b; S1 Table), indicating that *TPP* gene family in peanut may have important regulatory roles in various biological processes.

### Collinearity analysis of *TPP* family genes within the peanut genome and homologous relationships between peanut and rice, *Arabidopsis*, and soybean

**Fig 7:**
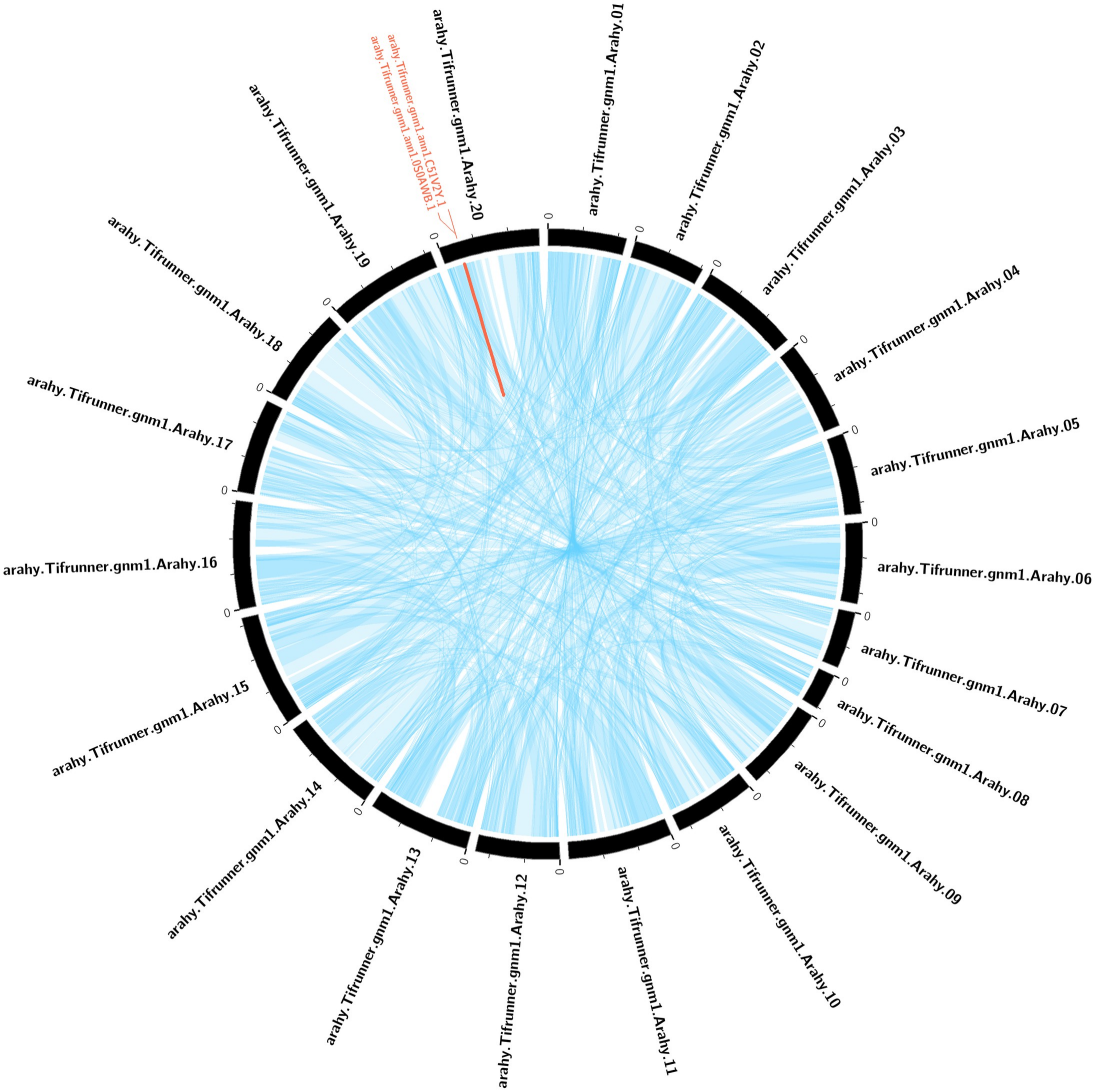
Inter-chromosomal relationships of genes in the peanut genome. Blue lines indicate all synteny blocks in the peanut genome, and the red line indicates tandemly duplicated *TPP* gene pairs.

The gene copies present on the genome serve as valuable references for gene evolution analysis. Segmental duplication and tandem duplication are the primary mechanisms responsible for the expansion of plant gene families [33]. The collinearity analysis within the peanut genome showed that only one pair of genes (*AhTPP42* and *AhTPP43*) in *TPP* gene family experienced tandem duplication on chromosome 20, with no segmental duplication events observed (Fig 7).

**Fig 8:**
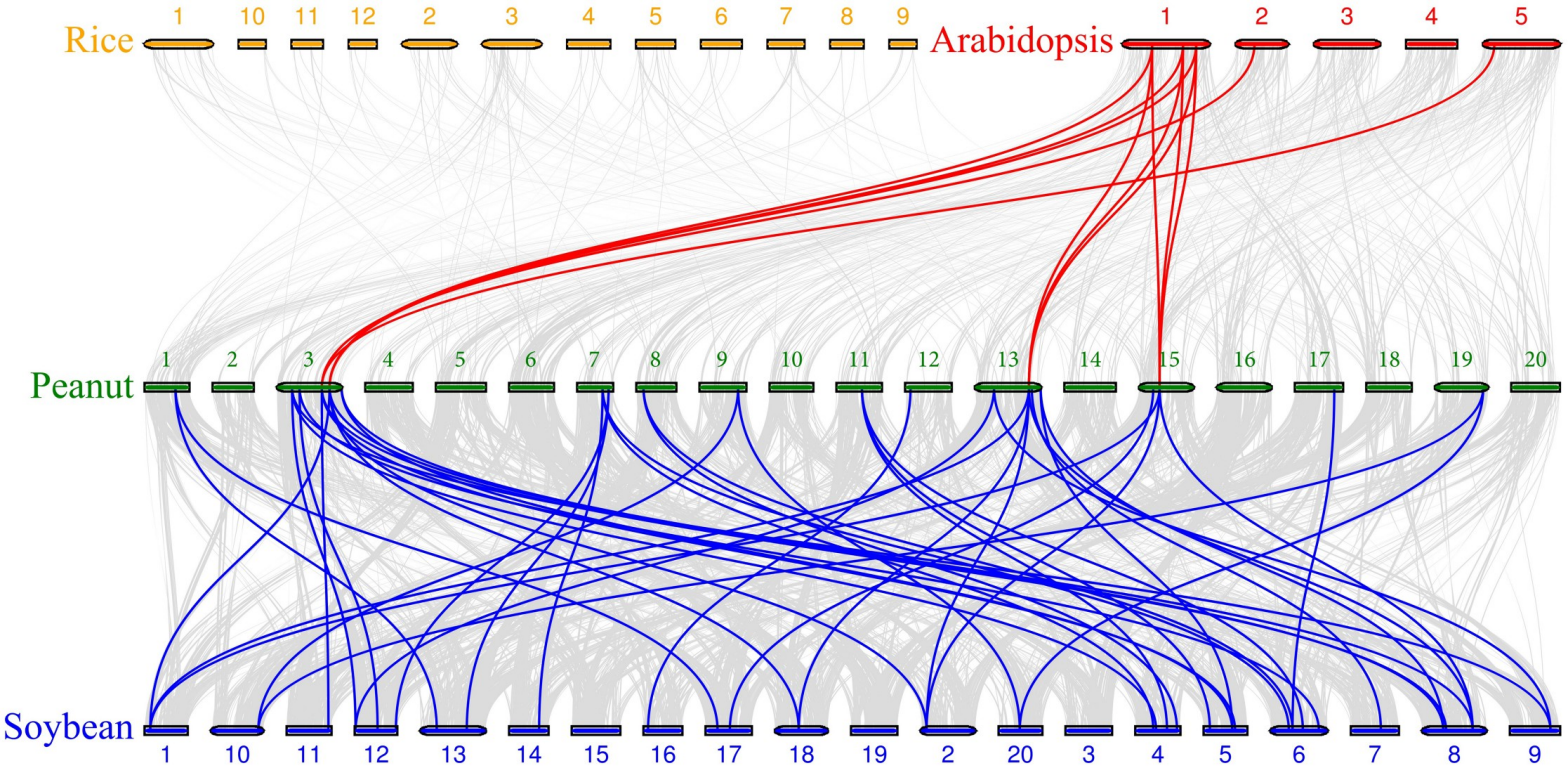
Collinearity relationship of *TPP* genes in peanut, soybean, rice, and *Arabidopsis*.

We constructed homologous maps of peanuts with three representative plants, including two dicotyledonous plants (*Arabidopsis thaliana*, *Glycine max*) and one monocotyledonous plant (*Oryza sativa* japonica). The results showed that no collinearity was detected between *AhTPP*s and rice genes, and 22 *AhTPP*s were collinear with genes from soybean (31) and *Arabidopsis* (9). The number of homologous gene pairs between these species was 45 and 11, respectively, suggesting that these homologous gene pairs were formed through gene replication during species differentiation (Fig 8; S2 Table).

### Expression pattern analysis of *TPP* gene family in peanut under low temperature, drought, and water-nitrogen colimitation conditions based on transcriptome data

**Fig 9:**
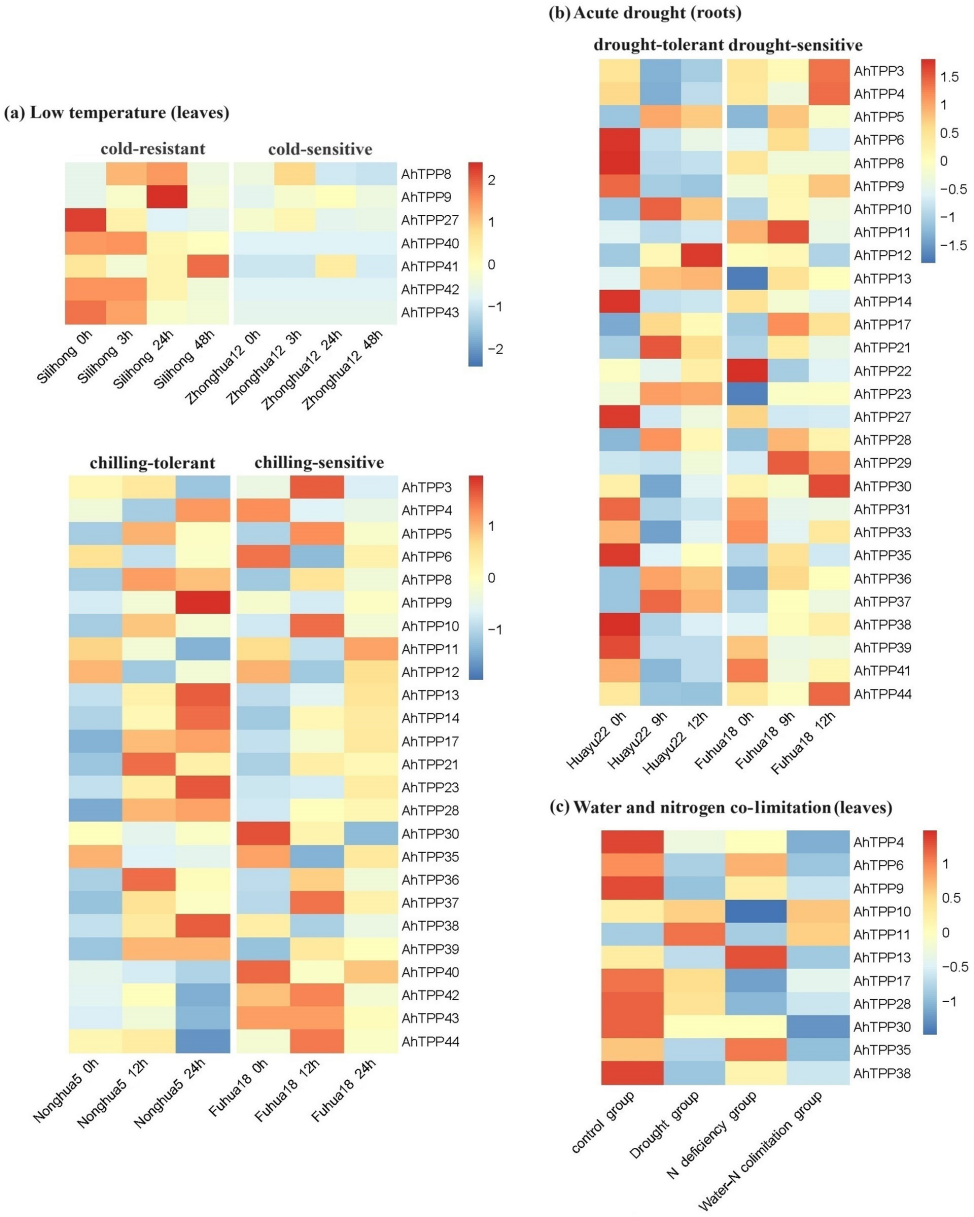
Expression pattern analysis of *AhTPP*s under low temperature, acute drought, and water-nitrogen co-limitation treatment based on publicly available transcriptome data.

We conducted expression pattern analysis of *TPP* gene family in peanut under low temperature, drought, and water-nitrogen colimitation conditions using four publicly available transcriptome datasets. Analysis has found that the accumulation of trehalose is of great significance for peanut to resist low-temperature damage. The low-temperature environment significantly induces the upregulation of most *TPP* gene family members in leaves, especially in cold-resistant varieties (Fig 9a; S3 Table). Drought stress significantly upregulates the expression of most *TPP* gene family members in roots, whereas their expression is inhibited in leaves (Fig 9b; S3 Table). Soil nitrogen deficiency inhibits the expression of *TPP* genes in leaves, and the inhibitory effect is particularly pronounced under the water-nitrogen colimitation condition (Fig 9c; S3 Table). Among them, significant increases were detected in the expression levels of *AhTPP13*, *AhTPP17*, *AhTPP21*, *AhTPP23*, and *AhTPP36* in cold-stressed leaves and drought-stressed roots, suggesting that they have regulatory effects on stress adaptation across diverse environmental conditions.

### Relative expression analysis of *TPP* family genes under salt stress in peanut by qRT-PCR

To further investigate the response of *TPP* gene family to salt stress in peanut, we utilized qRT-PCR to assess the relative expression levels of 45 *AhTPP*s. The information on primers for qRT-PCR analysis is listed in S4 Table.

**Fig 10:**
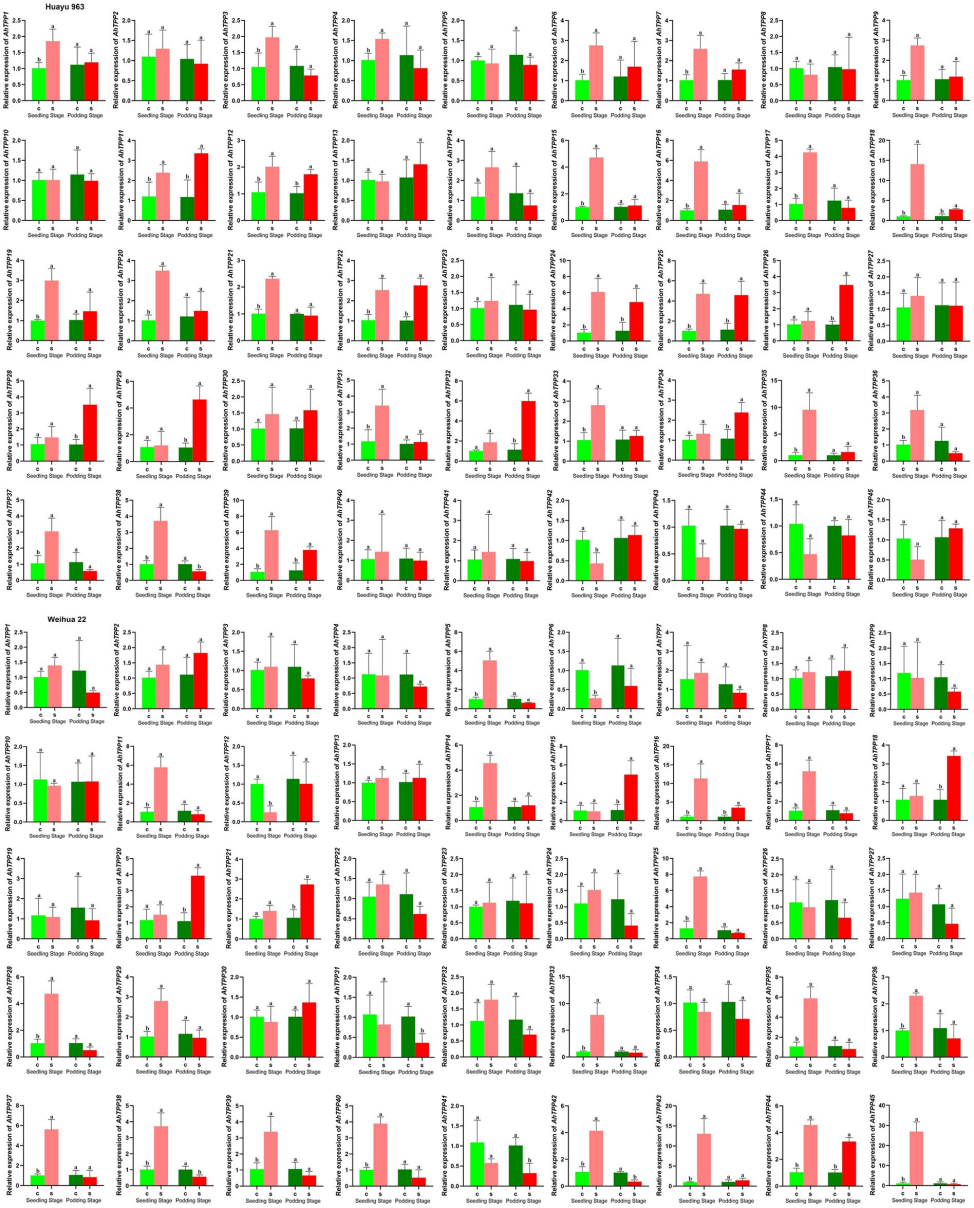
qRT-PCR analysis of *AhTPP*s under salt treatment during seedling stage and podding stage, respectively.

The results showed that most *AhTPP*s with differential expression under salt stress were upregulated. During the seedling stage of the salt-resistant variety (Huayu963), the relative expression levels of 24 *AhTPP*s were significantly upregulated, while during the podding stage, the relative expression levels of 10 *AhTPP*s were significantly upregulated. For the salt-sensitive variety (Weihua22), the relative expression levels of 19 *AhTPP*s were significantly upregulated during the seedling stage, while the relative expression levels of 6 *AhTPP*s were significantly upregulated during the podding stage, and the quantity was significantly lower than Huayu963 (Fig 10). Among them, some high expression genes were active, such as *AhTPP18*, *AhTPP25*, and *AhTPP39*, which were significantly upregulated under salt stress in two varieties and different growth stages (S3 Fig). It is speculated that they play a crucial positive role in promoting trehalose synthesis in peanut leaves to cope with soil salt damage.

## Discussion

### Trehalose and its precursor substances, which are vital components of the osmoregulatory system, significantly contribute to plant resistance to abiotic stress

Trehalose is a typical stress metabolite that effectively maintains the stability of cell membranes and macromolecules. When living organisms are growing under optimal conditions, they do not accumulate large amounts of trehalose. However, under stressful conditions such as nitrogen starvation, drought, extreme heat, and saline alkaline environments, trehalose accumulates rapidly [34, 35]. The chemical properties of trehalose are very stable, and it is not easy to decompose even under high temperatures and acidic conditions. At the same time, trehalose lacks reducing terminals related to the formation of glycosidic bonds, making it less prone to Maillard reactions. Trehalose’s ability to form a glassy structure allows it to stabilize proteins and lipids in membranes under water deficiency or low-temperature conditions, indicating that trehalose has a strong anti-dehydration effect and protects biological structures from abiotic stress damage such as high temperature, drought, and high salinity [36, 37].

Despite the extremely low levels of trehalose in higher plants, recent studies have demonstrated that trace amounts of trehalose and its precursor T6P are involved in a series of physiological processes such as embryonic development, flower induction, senescence regulation, seed filling, and response to biotic and abiotic stress in plants [38–41]. Peanut can improve the adaptability to salt stress by regulating a series of secondary metabolic processes, and significantly affect the expression of core genes in the process of flavonoids biosynthesis, phenylpropanoid biosynthesis, starch and sugar metabolism, nitrogen metabolism, circadian rhythm and so on [42]. Previous studies on the metabolomic data of leaves from two peanut genotypes under salt stress have confirmed that trehalose-6-phosphate and D-trehalose are important differential metabolites, indicating that trehalose biosynthesis plays an active role in the response of peanut leaves to salt stress [43].

The sugar signaling pathway significantly impacts resistance to abiotic stress in plants, with the T6P pathway serving as a crucial component. This pathway not only participates in stress signal transduction but also regulates secondary metabolite production [44]. The intermediate product T6P, which is involved in the synthesis of trehalose, plays an important role in regulating the sucrose content and transportation of plants [45]. It has been suggested in several studies that changes in T6P content enhance plant stress resistance in the trehalose metabolism pathway, rather than trehalose itself. T6P can interact with Sucrose non-fermenting related kinase-1(SnRK1) to regulate the allocation and utilization of plant carbon, enabling plants to survive in adverse environments [46]. As an important osmotic regulator, trehalose maintains the osmotic potential in plants to resist salt-alkali stress. Our study also confirmed that the trehalose content in peanut leaves under salt stress was higher than that in the control treatment, and reached a significant level in the seedling stage of both peanut varieties, indicating that the accumulation of trehalose has a positive significance for the survival of peanuts in saline-alkali environments.

### Identification, structural analyses, and function prediction of *TPP* gene family in peanut

A total of 45 *AhTPP*s were identified in this study. Based on amino acid sequences, phylogenetic relationships, and motif analysis, these 45 *AhTPP*s can be divided into three subgroups. The majority of *AhTPP*s have TPS domains at the N-terminal and TPP domains at the C-terminal, and all *AhTPP*s have at least one TPP domain. This characteristic is consistent with previous research findings in *Arabidopsis*, rice, and *Brachypodium distachyon* [24, 25, 47]. Among them, the average length of the 8 *AhTPP*s in the third subgroup is the shortest, and they have a distant genetic relationship with *TPP* genes of other species, suggesting that they may play unique functions in peanut. The first and second subgroups also have different characteristics. The number of introns among the members of the first subgroup varies greatly, with the highest being close to 20 and the lowest being only 2. It is also the subgroup with the richest types of motifs, with most members containing 10 different motifs. The number of introns among the members of the second subgroup is relatively consistent, ranging from 5 to 10, but there are fewer types of motifs, only appearing motif 3, motif 7, motif 9, and motif 10. The different characteristics between these two subgroups indicate that they may have different biological functions.

Each member of a gene family exhibits similar gene structures and functions, potentially as a result of expansion through species genome replication events [25, 48]. Compared to other eukaryotes, plants typically have a higher rate of genome replication [49]. Previous studies have identified 10, 13, and 26 *TPP* genes in *Arabidopsis*, rice, and maize, respectively [24, 25, 50]. The *TPP* gene family has significant differences in quantity among different species, indicating that this family may undergo gene duplication during plant evolution. The number of gene family members is related to the size of the species genome, and the larger the genome, the higher the probability of duplication events occurring. In *Arabidopsis*, 10 *TPP* genes have been identified, of which 8 are paired homologous genes [25, 51], while allopolyploid plants such as wheat seem to retain copies of all *TPP* genes from their ancestral species [16]. This study detected a tandem duplication event on chromosome 20 of peanut, suggesting that tandem duplication also plays a role in the expansion of *TPP* gene family.

The characteristics of gene response are closely related to the cis-acting elements of the promoter. Almost all promoters of *AhTPP*s contain cis-acting elements related to light response. All *AhTPP*s contain a large number of hormone-responsive elements. Among them, more than half of the gene family members contain methyl jasmonate responsive elements, with *AhTPP6*, *AhTPP7*, *AhTPP20*, *AhTPP34*, and *AhTPP45* being more prominent. Methyl jasmonate plays an important role in plant adaptation to low temperatures and resistance to pests [52, 53]. Some members also contain salicylic acid-responsive elements, which can enhance the drought resistance of plant seedlings and promote seed germination and seedling growth under salt stress to a certain extent [54]. The number of abscisic acid-responsive elements in *AhTPP12* is the highest, suggesting that it may be involved in ABA signal response. In terms of stress response, more than half of the gene family members contain drought, low temperature, and anaerobic response elements, suggesting that the *TPP* gene family plays an important role in peanut resistance to abiotic stress. The enhancement of phenolic metabolism in plants caused by mechanical damage is a physiological response of plants to accelerate wound healing. Some members contain wound response elements, which may play a certain role in peanut wound recovery.

### *TPP* genes enhance stress resistance by regulating trehalose synthesis in plants

By inducing the expression of *TPP* genes to regulate the trehalose metabolism pathway, it is possible to increase the trehalose content in plants, thereby enhancing their tolerance to various abiotic stresses. According to research, the expression levels of *TPS* and *TPP* genes in *Arabidopsis* are positively correlated with trehalose content under high temperature stress [55, 56]. The 10 members of *TPP* gene family in *Arabidopsis* have different spatiotemporal expression patterns and stress response patterns. Among them, *AtTPPD* is involved in regulating sugar metabolism under salt conditions, and overexpression leads to an accumulation of starch and soluble sugars. It is currently the only gene in *TPP* gene family of *Arabidopsis* known to be related to salt stress [11]. Overexpression of *AtTPPF* increased the content of trehalose, sucrose, and soluble sugars under drought conditions, while upregulating the expression levels of drought responsive genes [57]. Among the 13 *TPP* genes of rice, overexpression of *OsTPP1* increased endogenous trehalose content and improved the survival rate of rice under low temperature stress [24]. The expression of *OsTPP2* is regulated by stress such as low temperature, drought, salt, and ABA [58]. Overexpression of *OsTPP3* enhanced the tolerance of rice plants to PEG stress, and gene expression analysis showed that the expression levels of genes related to stress response and ABA synthesis were upregulated [17]. Overexpression of *OsTPP7* increased the germination rate of seeds under anaerobic conditions and improved the seedling rate of rice direct seeding [59].

The response of genes involved in the trehalose metabolism pathway of plants to abiotic stress is closely associated with the presence of inducible cis-acting elements in their promoters’ regions. Research has shown that the anaerobic responsive elements ARE and GC-motif of *OsTPP3* are responsible for its high expression during seed germination under hypoxic conditions [60]. Under high temperature stress, the expression of *RcTPS7b* in rose significantly increased, and there were multiple ABA responsive elements in the promoter of this gene. It is speculated that *RcTPS7b* may be involved in the ABA signaling pathway for heat stress resistance [61]. *CsTPP*s’ promoters are rich in various cis-acting elements, which may be involved in response processes of abiotic stress such as low temperature and drought in tea plants [62]. Our study also found that the promoter regions of *TPP* gene family members in peanut contain multiple stress-responsive and hormone-responsive elements. Hormone-responsive elements mainly include ABRE, ERE, P-box, CGTCA-motif, and TGACG-motif, while stress-responsive elements mainly include MYC, MBS, ARE, and STRE, further indicating that this gene family plays an important role in regulating peanut growth and development and stress resistance processes.

Various osmotic regulatory substances exist in plants, including betaine, proline, and soluble proteins. Overexpression of genes related to the trehalose metabolism pathway can increase the content of proline and soluble proteins, and improve plant salt tolerance [12, 63]. The deposition of lignin may serve as a hydrophobic barrier to prevent ions from entering the plant xylem and reduce the damage of salt stress to plant tissues [64]. Research has shown that the significant improvement in salt tolerance of transgenic rice lines overexpressing *OsTPS8* is mainly due to *OsTPS8*’s function to promote lignin deposition in rice through ABA signaling [65].

Our study analyzed the transcriptome data of the peanut *TPP* gene family and found that most *AhTPP*s have a significant response to low temperature and drought stress. Furthermore, we employed quantitative real-time PCR to analyze the expression patterns of *AhTPP*s under salt stress and identified several genes that are responsive to salt stress. We found that the expression levels of most members of the peanut *TPP* gene family increased to varying degrees under salt stress. Among them, *AhTPP18*, *AhTPP25*, and *AhTPP39* were significantly upregulated in both varieties and exhibited a long-lasting response. It is speculated that these genes regulate trehalose synthesis and play an important role in peanut’s resistance to salt stress.

Salt tolerance of plants is a complex quantitative trait controlled by multiple genes [66]. During the response to salt stress, a large number of genes are activated, leading to the accumulation of many secondary metabolites involved in stress resistance, including trehalose, which are regulated by specific TFs [67]. At present, there are many studies on physiological changes of cultivated peanut under salt stress, but little attention has been paid to trehalose in the response of peanut to salt stress, and the research on its molecular mechanism is mostly focused on a few independent genes, so it is not easy to obtain systematic genetic information against salt stress. In the future, we plan to screen the key genes regulating trehalose biosynthesis under salt stress through the joint analysis of multiomics, combined with the currently identified active members of *TPP* gene family in peanut, and clarify their biological functions and regulatory networks by gene editing, in order to provide more insights into the molecular mechanism of *AhTPP*s regulating trehalose accumulation and improving salt tolerance of peanut.

## Conclusion

This study conducted the genome-wide identification of *TPP* gene family in peanut and analyzed its expression patterns under salt stress. A total of 45 candidate *TPP* genes were identified in the peanut genome. Phylogenetic analysis showed that *AhTPP*s have highly conserved motifs and gene structures. Collinearity analysis suggested that tandem duplication may have played a role in the expansion of *TPP* gene family evolution in peanut. The analysis of cis-acting elements in the promoter regions of *AhTPP*s indicated that most of them are involved in various stress responses, providing a basis for the functional research of *AhTPP*s. Transcriptome data showed that *AhTPP*s respond to abiotic stress such as low temperature, drought, and nitrogen deficiency. In addition, the expression of *AhTPP18*, *AhTPP25*, and *AhTPP39* was significantly upregulated under salt stress. The specific regulatory mechanisms of *TPP* gene family in peanut growth, development, and stress adaptation should be further elucidated through genetic transformation experiments.

## Supporting information

S1 Fig Phenotypes of the salt-sensitive variety Weihua22 and the salt-resistant variety Huayu963 during seedling stage under salt treatment (S) and normal cultivation treatment (C). (DOCX)

S2 Fig Correlation analysis among trehalose content, the maximal quantum yield of PSII (Fv/Fm), net photosynthetic rate (Pn), stomatal conductance (Cond), actual photochemical efficiency of PSII (ΦPSII), and chlorophyll content (SPAD) of control and salt stress treatments at the seedling stage of peanut. (DOCX)

S3 Fig Venn diagram of significantly upregulated *AhTPP*s under salt stress for two peanut varieties and two growth stages. (DOCX)

S1 Table The distribution of cis-acting elements in promoters of the *TPP* gene family members in peanut. (XLSX)

S2 Table Collinear analysis of TPP genes between *Arachis hypogaea*, *Arabidopsis thaliana*, and *Glycine max*. (XLSX)

S3 Table The FPKM values of *AhTPPs* in transcriptome data under low-temperature, acute drought, and water-nitrogen co-limitation stress. (XLSX)

S4 Table List of primers used in qRT-PCR analysis. (XLSX)

S1 File (FA)

S2 File (FA)

S3 File (FA)
