# Supplementary material for: Genome-wide identification and expression analysis of TPP gene family under salt stress in peanut (Arachis hypogaea L.)
Source: PLoS One. 2024 Jul 18;19(7):e0305730. doi: 10.1371/journal.pone.0305730 (PMC11257338; doi:10.1371/journal.pone.0305730)
Supplement: S2 Fig — (DOCX) [file pone.0305730.s002.docx]

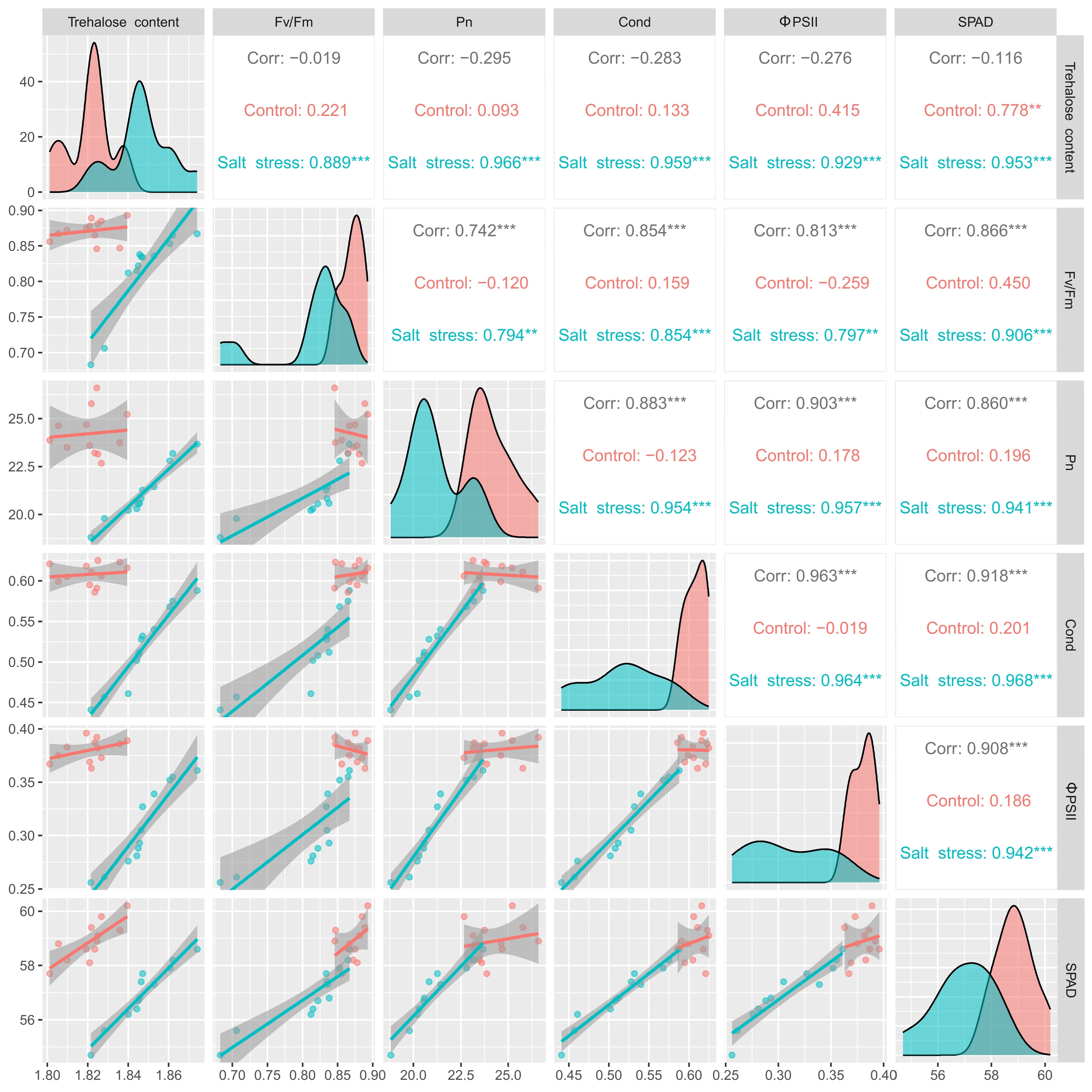


**Fig. S2.** Correlation analysis among trehalose content, the maximal quantum yield of PSII (Fv/Fm), net photosynthetic rate (Pn), stomatal conductance (Cond), actual photochemical efficiency of PSII (ΦPSII), and Chlorophyll content (SPAD) of control and salt stress treatments at the seedling stage of peanut.
