# Supplementary material for: Genome-wide identification and expression analysis of TPP gene family under salt stress in peanut (Arachis hypogaea L.)
Source: PLoS One. 2024 Jul 18;19(7):e0305730. doi: 10.1371/journal.pone.0305730 (PMC11257338; doi:10.1371/journal.pone.0305730)
Supplement: S3 Fig — (DOCX) [file pone.0305730.s003.docx]

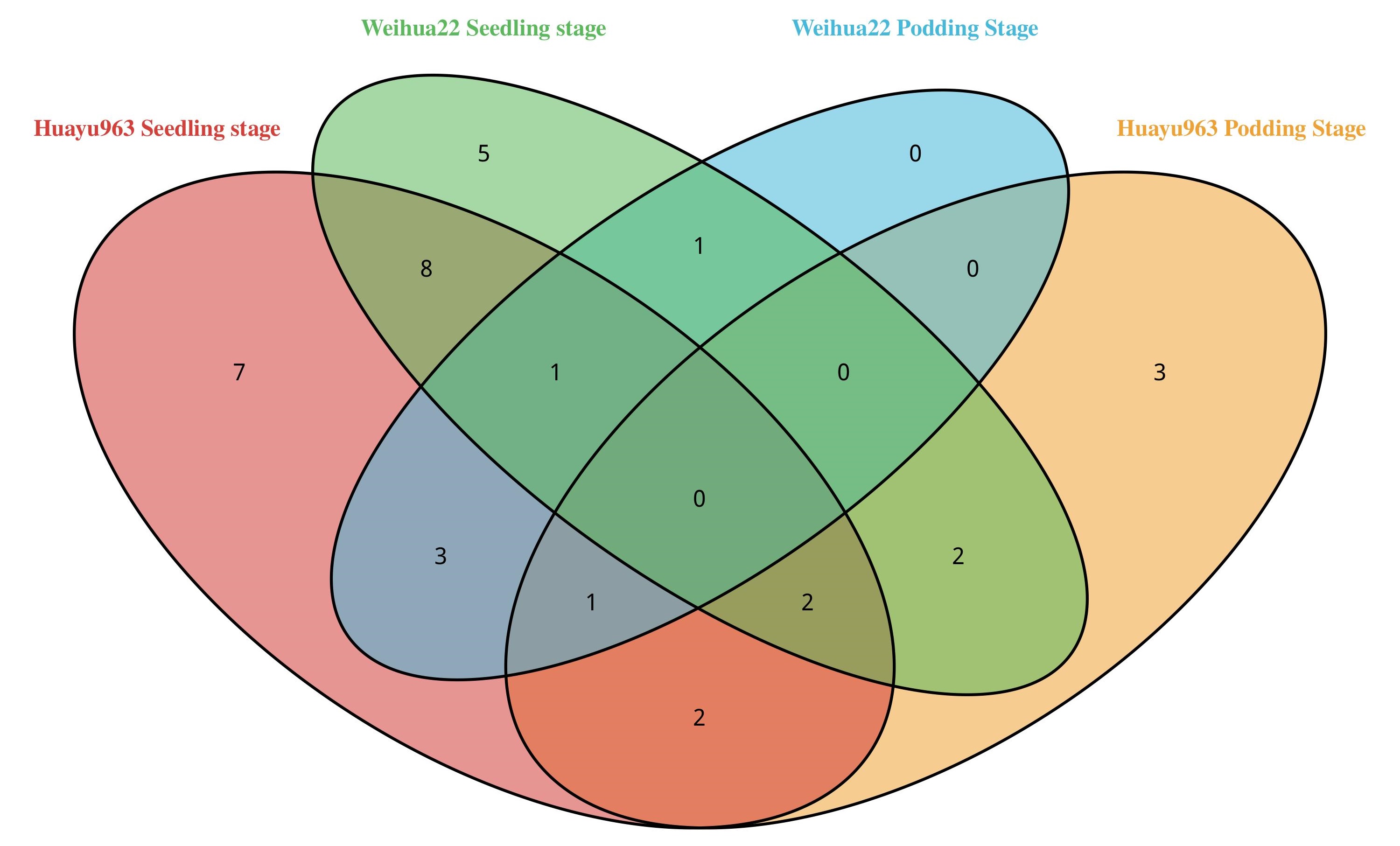


**Fig. S3.** Venn diagram of significantly upregulated *AhTPP*s under salt stress for two peanut varieties and two growth stages.
